# Gene Therapy of Multiple Sclerosis Using Interferon ****β****-Secreting Human Bone Marrow Mesenchymal Stem Cells

**DOI:** 10.1155/2013/696738

**Published:** 2013-04-22

**Authors:** Chung Heon Ryu, Kwang Ywel Park, Yun Hou, Chang Hyun Jeong, Seong Muk Kim, Sin-Soo Jeun

**Affiliations:** ^1^Postech-Catholic Biomedical Engineering Institute, Seoul St. Mary's Hospital, The Catholic University of Korea, 505 Banpo-daero, Seocho-gu, Seoul 137-701, Republic of Korea; ^2^Department of Biomedical Science, College of Medicine, The Catholic University of Korea, 505 Banpo-daero, Seocho-gu, Seoul 137-701, Republic of Korea; ^3^Department of Neurosurgery, Seoul St. Mary's Hospital, The Catholic University of Korea, 505 Banpo-daero, Seocho-gu, Seoul 137-701, Republic of Korea

## Abstract

Interferon-beta (IFN-**β**), a well-established standard treatment for multiple sclerosis (MS), has proved to exhibit clinical efficacy. In this study, we first evaluated the therapeutic effects for MS using human bone marrow-derived mesenchymal stem cells (hBM-MSCs) as delivery vehicles with lesion-targeting capability and IFN-**β** as therapeutic gene. We also engineered hBM-MSCs to secret IFN-**β** (MSCs-IFN**β**) via adenoviral transduction and confirmed the secretory capacity of MSCs-IFN**β** by an ELISA assay. MSCs-IFN**β**-treated mice showed inhibition of experimental autoimmune encephalomyelitis (EAE) onset, and the maximum and average score for all animals in each group was significantly lower in the MSCs-IFN**β**-treated EAE mice when compared with the MSCs-GFP-treated EAE mice. Inflammatory infiltration and demyelination in the lumbar spinal cord also significantly decreased in the MSCs-IFN**β**-treated EAE mice compared to PBS- or MSCs-GFP-treated EAE mice. Moreover, MSCs-IFN**β** treatment enhanced the immunomodulatory effects, which suppressed proinflammatory cytokines (IFN-*γ* and TNF-*α*) and conversely increased anti-inflammatory cytokines (IL-4 and IL-10). Importantly, injected MSCs-IFN**β** migrated into inflamed CNS and significantly reduced further injury of blood-brain barrier (BBB) permeability in EAE mice. Thus, our results provide the rationale for designing novel experimental protocols to enhance the therapeutic effects for MS using hBM-MSCs as an effective gene vehicle to deliver the therapeutic cytokines.

## 1. Introduction

Multiple sclerosis (MS) is an autoimmune-mediated inflammatory disease in which the myelin sheaths around the axons of the central nervous system (CNS) are damaged, leading to demyelination and neuronal loss [1]. MS is characterized by multiple signs and symptoms, with remissions and recurring exacerbations of the disease. A number of experimental treatments have been developed to improve the survival for patients with MS. There are several approved therapies for MS, such as glatiramer acetate and mitoxantrone, which mainly target the immunological aspects [2, 3]. Although there are several approved drugs for MS, many patients do not respond optimally to these drugs. Therefore, developing more effective therapeutic protocols for MS is necessary. Experimental allergic encephalomyelitis (EAE) is an animal model of CNS autoimmune disease that follows immunization with certain CNS antigens. The EAE model has been widely used as a human MS model, and many clinical and histopathological similarities to human MS have been reported [4].

Mesenchymal stem cells (MSCs) are adult multipotent cells that differentiate into the mesenchymal lineages of adipocytes, osteocytes, and chondrocytes. Currently, MSCs are investigated in preclinical and clinical settings because of their self-renewal capacity, ability to differentiate into multiple lineages, and immunosuppressive activity [5, 6]. Moreover, MSCs can migrate to areas of injury [7]. This pathotropism of MSCs makes them useful for the regeneration of damaged tissues as well as for targeted delivery of therapeutic genes to sites of pathology. Recently, allogenic human MSCs have been proposed for the treatment of autoimmune diseases [8]. For example, human bone marrow-derived MSCs (hBM-MSCs) improved functional recovery in both chronic and relapsing-remitting models of mouse EAE, traced their migration into the injured CNS, and assayed their ability to modulate disease progression and the host immune response [9]. Additionally, human bone marrow stromal cell treatment also improved functional recovery after EAE in mice, possibly, via reducing inflammatory infiltrates and demyelination areas, stimulating oligodendrogenesis, and by elevating BDNF expression [10]. These data indicated that hBM-MSCs could be used as the promising delivery vehicle of therapeutic gene against EAE.

Interferon-beta (IFN-*β*) as one of the promising treatments for MS is approved for the treatment of relapsing-remitting MS. IFN-*β* treatments have been shown to produce about 18%–38% reduction in the rate of MS relapses and to slow the progression of disability in MS patients [11]. Its exact mechanism of action for MS therapy is unknown but probably includes the regulation of T-cell activation and immune cell proliferation, autoreactive T-cell apoptosis, IFN-*γ* antagonism, modulation of proinflammatory (Th1)/anti-inflammatory (Th2) cytokines, and inhibition of immune cell trafficking across the blood-brain barrier (BBB) [12]. Despite the impressive therapeutic effects in the experimental studies for MS, clinical trials using IFN-*β* treatment have poor outcome [11]. One of the main problems is the insufficient duration of recombinant IFN-*β*, short half-life *in vivo*, and its inaccessibility to the CNS. A second problem is the presence of BBB, preventing many therapeutics, especially large and/or charged molecules, from reaching the brain and spinal cord to treat disorders of the CNS [13]. To overcome these problems, we used hBM-MSCs as IFN-*β* gene delivery vehicles with CNS targeting migration capabilities and evaluated the therapeutic efficiency of IFN-*β*-secreting hBM-MSCs (MSCs-IFN*β*) in EAE.

## 2. Materials and Methods

### 2.1. Mesenchymal Stem Cell Culture and Adenovirus Infection

MSCs derived from the human bone marrow were purchased from Lonza (Walkersville, Maryland, USA). The MSCs were subcultured at a concentration of 5 × 10^4^ cells/cm^2^ in MSC growth medium (Lonza) and used for experiments during passages 5 to 8. The recombinant adenoviral vector encoding the gene for EGFP (Ad-GFP) and mouse IFN-*β* (Ad-IFN-*β*) was constructed and produced using the AdEasy vector system, following the manufacturer's instructions (Quantum Biotechnologies, Carlsbad, CA, USA). MSCs were infected with 50 multiplicity of infection of Ad-GFP or Ad-IFN-*β*, as described previously [14].

### 2.2. EAE Induction and Treatments

EAE was induced in female C57BL/6 mice (10 weeks old, Charles River Laboratories) using immunization with MOG35–55. The mice were injected subcutaneously at two sites with a total of 200 *μ*g of MOG35–55 emulsified in complete Freund's adjuvant (CFA) containing 6 mg/mL of Mycobacterium tuberculosis. Two h and twenty four h after the MOG35–55 injection, the mice received 100 ng *Bordetella pertussis* toxin intraperitoneally, respectively. Mice were scored as follows: 0: no clinical signs; 1: limp tail; 2: partial hind leg paralysis; 3: complete hind leg paralysis; 4: complete hind leg paralysis and partial front leg paralysis; and 5: moribund or dead. Mice were randomly divided into three groups and were injected intravenously (IV) on day 7 after immunization: PBS (*n* = 7), MSCs-GFP (*n* = 7, 1 × 10^6^ cells/each mouse), and MSCs-IFN*β* (*n* = 7, 1 × 10^6^ cells/each mouse).

### 2.3. Histological Analysis of Demyelination and Inflammatory Infiltration

Histological evaluation was performed on 4% paraformaldehyde fixed, O.C.T-embedded sections of lumbar spinal cords of EAE mice at day 37 after immunization. Frozen sections were stained with luxol fast blue (LFB) and hematoxylin-eosin (H&E) for evaluating inflammatory infiltration and demyelination, respectively. Quantification was performed on 3 sections per animal and 4 animals per group. The infiltration cells were counted by investigators blinded to the status of the animal. The average of infiltrated cell number and the intensity of demyelinated axons was determined from randomly selected areas within the lesion areas from at least 3 sections taken from the same spinal cord level from 4 animals in each group. All images were measured via a computer using the MetaMorph software (Molecular Devices, Downingtown, PA, USA).

### 2.4. Determination of Cytokines by ELISA and ELISPOT

Serum was obtained from three animals for each treatment group at day 37 after immunization. ELISA was performed to measure the concentrations of IFN-*γ*, TNF-*α*, IL-4, and IL-10 using Quantikine immunoassay kits from R&D systems (Minneapolis, MN). Enzyme-linked immunospot (ELISPOT) kit for the detection the IFN-*γ* and IL-4 was purchased from R&D systems. Briefly, the plates were blocked for 1 h with sterile PBS, containing 1% BSA, and washed 3 times with sterile PBS. Splenocytes (5 × 10^5^/well) isolated from each treatment group and incubated with MOG35–55 (10 *μ*g/mL) at 37°C for 48 h and then washed. Biotinylated antibody, streptavidin-AP, and NBT/5-bromo-4-chloro-3-indolyl phosphate substrate (Vector Labs, Burlingame) were used to detect IFN-*γ* and IL-4 secretion. Spots were counted using the Zeiss Microscopy (Oberkochen, Germany).

### 2.5. Reverse Transcription Polymerase Chain Reaction (RT-PCR)

Total RNA was prepared from the cerebral cortex using TRIzol (Invitrogen, Grand Island, NY), according to the manufacturer's instructions. cDNA was synthesized using 2 *μ*g total RNA and oligo(dT) primer and Superscript II polymerase for reverse transcription PCR (Invitrogen). PCR amplifications consisted of a total of 32 cycles of denaturation at 94°C for 30 sec, annealing at 54°C for 30 sec, extension at 72°C for 1 min with a first denaturation at 94°C for 7 min, and final extension at 72°C for 7 min. Target primers sequences used were as follows: BDNF forward: 5′-TGTG ACAGTAT TAGCGAGT GGGT-3′; BDNF reverse: 5′-ACGATTGGGTAGTTCGGCATT-3′; NGF forward: 5′-ACTCTGTCCCTGAAGCCCACTG-3′; NGF reverse: 5′-TGTTGCGGGTCTG CCCTGTC-3′; and GAPDH forward: 5′-TCCATGACAACTTTGGTATCG-3′; GAPDH reverse: 5′-TGTAGCCAAATTCGTTG TCA-3′.

### 2.6. *In Vivo* Fluorescence Imaging Analysis

IV-injected near infrared (NIR)-labeled MSCs-IFN*β* were tracked along time using the CRi Maestro *in vivo* imaging system (CRI, Inc., Woburn, MA), which allows the detection of labeled cells in each animal tissue. In brief, mice were anesthetized by gas mixtures of 1.5% isoflurane and air and then imaged. The biofluorescence signals (photons/sec) emitted from the mice were captured by a high-sensitivity charge-coupled device camera and analyzed using the Maestro software. Spectral unmixing algorithms were applied to create unmixed images of fluorescein and autofluorescence. To validate the sensitivity and specificity of this method, NIR-labeled MSCs-IFN*β* in the brain and spinal cord were detected by immunofluorescence staining using an anti-hNA (human nuclear antigen) polyclonal antibody (Molecular Probes, Eugene, OR), followed by detection with an anti-rabbit secondary antibody conjugated with Alexa 488 (Molecular Probes).

### 2.7. Fluorescent Detection of Evans Blue Dye

The uptake of the Evans blue (EB) tracer marker from circulation into the cerebrum and spinal cord tissues was measured using a spectrophotometer. Briefly, 4% EB was infused by IV injection in the tail vein in each mouse in the control groups as well as individual mice identified from the experimental EAE groups at the peak of clinical disease. After 4 hours, the mice were sacrificed by transcardiac perfusion with PBS, and the spinal cords removed and homogenized in 0.5% TritonX-100. The supernatants were plated 100 *μ*L/well in triplicate in a flat-bottom 96 well plate, and fluorescence was quantified by using a microplate fluorescence reader (PerkinElmer, Wellesley, MA, USA), (excitation: 620 nm; emission: 680 nm). Values are presented as intensity of fluorescence and represent average values for at least three mice per group.

### 2.8. Statistical Analysis

All data are expressed as the mean ± SEM. Statistical differences between different test conditions were determined using Student's *t*-test. Probability values less than 0.05 were considered significant.

## 3. Results and Discussion

### 3.1. Clinical Status of IFN-*β* Secreting MSCs Transplanted Mice after EAE Induction

**Figure 1:**
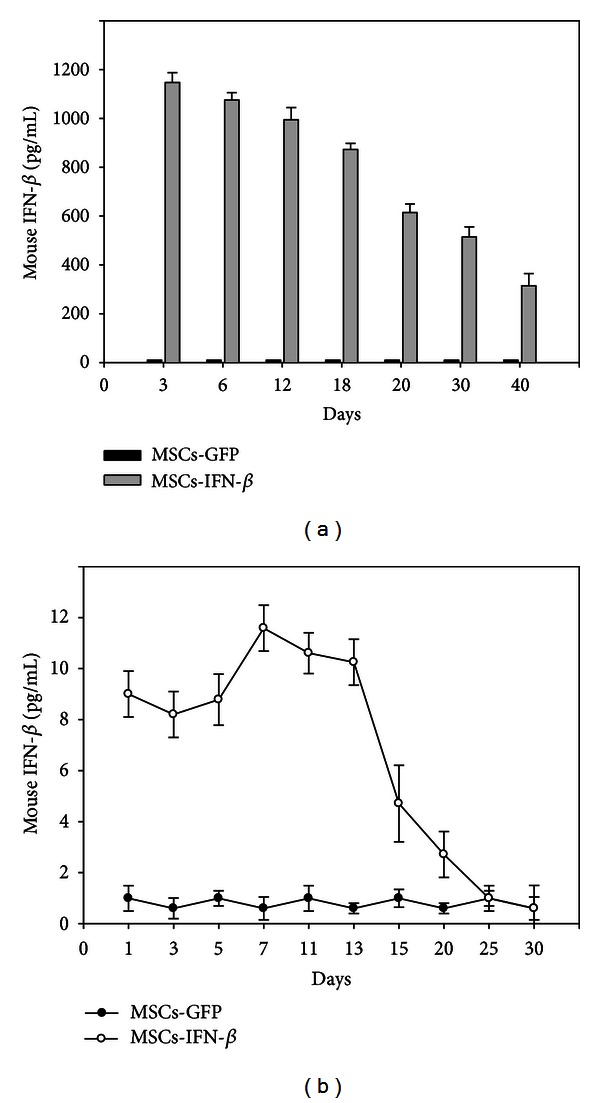
*In vitro* and *in vivo* transgene expression for MSCs-IFN*β*. (a) IFN-*β* protein production was detected in supernatants from IFN-*β*-transduced MSCs by ELISA. MSCs-GFP was used as a control. (b) For the quantification of secreted IFN-*β* and longevity of IFN-*β* expression in EAE, serum was isolated on different days (*n* = 3/each group) after treatment and then assessed by ELISA. Columns: mean; bars: SE. The results are representative of three independent experiments.

**Figure 2:**
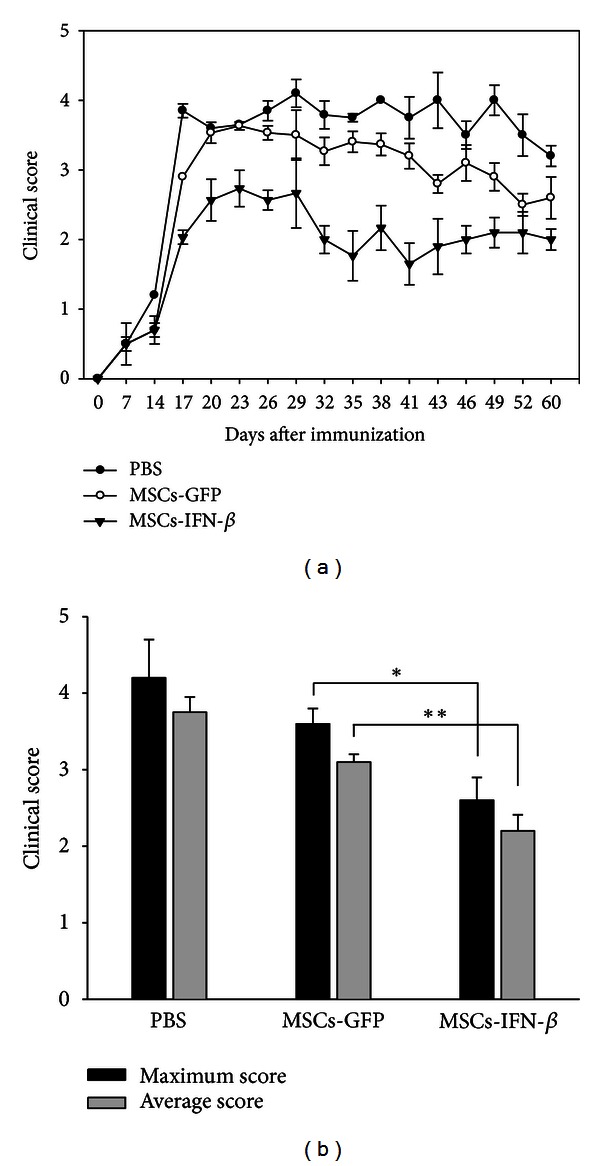
Clinical severity of EAE mice treated with MSCs-IFN*β*. (a) EAE was induced in female C57 BL/6 mice by the immunization with the MOG35–55. After day 7, immunized mice were either treated with PBS, MSCs-GFP, or MSCs-IFN*β*, and the MSCs-IFN*β* group (*n* = 7/each group) resulted in a significant amelioration of clinical symptoms. (b) The maximum and average scores for all animals in the PBS-, MSCs-GFP- and MSCs-IFN*β*-treated EAE mice over the 60-day period (*n* = 7/each group). Columns: mean; bars: SE. *P < 0.05, **P < 0.01, and Student's *t*-test. The results are representative of three independent experiments.

In this study, we first used hBM-MSCs as vehicles to deliver IFN-*β* gene for MS therapy. hBM-MSCs have a number of advantages over other stem cell types. It can be easily obtained from human bone marrow upon bone biopsy, expand *in vitro*, and can inhibit the proliferation of T, B, and dendritic cells through the induction of cell division arrest. hBM-MSCs can also inhibit the proliferation of natural killer cells and impair antigen presentation. Moreover, allogenic human MSCs have been recently proposed for the treatment of acute graft-versus-host disease (GVHD) [8]. Thus, immunomodulatory capacities of hBM-MSCs may also provide a synergistic therapeutic effect for EAE. MS is a chronic inflammatory disorder which usually requires long-term treatment. Recombinant IFN-*β* (rIFN-*β*) is currently the most common therapy for MS. At present, two IFN-*β* drugs (IFN-*β*1b and IFN-*β*1a) have been approved for treatment of MS: IFN-*β*1b 250 mg subcutaneous (s.c.) every other day, IFN-*β*1a 22 mg s.c. three times per week, and IFN-*β*1a 30 mg intramuscular (i.m.) once a week [15]. Administration of rIFN-*β* is typically associated with peak and trough kinetics governed by the biologic half-life of the protein. IFN-*β* has a very short half-life in the serum. Moreover, long-term repeated injections of rIFN-*β* are associated with several clinically relevant side effects, including depression, inflammation, and liver toxicity [16]. In this experiment, IFN-*β*-transduced MSCs (MSCs-IFN*β*) could provide a long-term expression of IFN-*β* at therapeutic concentration. To identify whether MSCs-IFN*β* can secrete IFN-*β* protein *in vitro* and injected MSCs-IFN*β* survived *in vivo* for a sufficient length of time to allow a therapeutic effect to occur, we measured the levels of IFN-*β* protein and its longevity in EAE mice serum. IFN-*β* in supernatants from MSCs-IFN*β* was detected from day 3 and persisted to day 40. IFN-*β* in supernatants from GFP-transduced MSCs (MSCs-GFP) as control could not be detected with this method (Figure 1(a)). ELISA analysis on different days after MSCs-IFN*β* treatment in EAE also revealed that the IFN-*β* protein expressed in serum peaked on day 7, began to decrease after day 13, and persisted for 3 weeks (Figure 1(b)). Additionally, IFN-*β* protein was detected in the serum of mice transplanted with MSCs-IFN*β* at higher levels than those expressed in mice transplanted with MSCs-GFP. The low level of IFN-*β* protein in the serum of mice transplanted with MSCs-GFP as control could be due to the induction by the MSCs transplantation, as MSCs are known to induce the secretion of beneficial cytokines by host cells [17]. Thus, a single administration of MSCs-IFN*β* could offer significant advantages over protein delivery by requiring less frequent injections and long-term expression of protein, resulting in therapeutic efficacy, while minimizing dose-limiting side effects. Next, we tested the therapeutic efficacy of MSCs-IFN*β* treatment on EAE mice. The therapy was started at the day 7 after immunization when the first mouse showed neurological symptoms. The mean clinical scores of each group were shown in Figure 2(a). We also evaluated the mean maximum and average score achieved by each animal from day 0 to day 40 after immunization. We observed that within the first 14 days of MSCs-IFN*β* administration, the neurological deficits were stabilized, and the maximum and average score for all animals in each group was significantly lower in the MSCs-IFN*β*-treated EAE mice when compared with the MSCs-GFP-treated EAE mice (*P* < 0.01) (Figure 2(b)). Taken together, these results indicate that MSCs could be used as a promising delivery vehicle for IFN-*β* gene, and, besides, MSCs-IFN*β* exhibit strong therapeutic effect via long-term secretion of IFN-*β* in EAE.

### 3.2. MSCs-IFN*β* Reduced Inflammation and Demyelination of Lumbar Spinal Cords in EAE Mice

**Figure 3:**
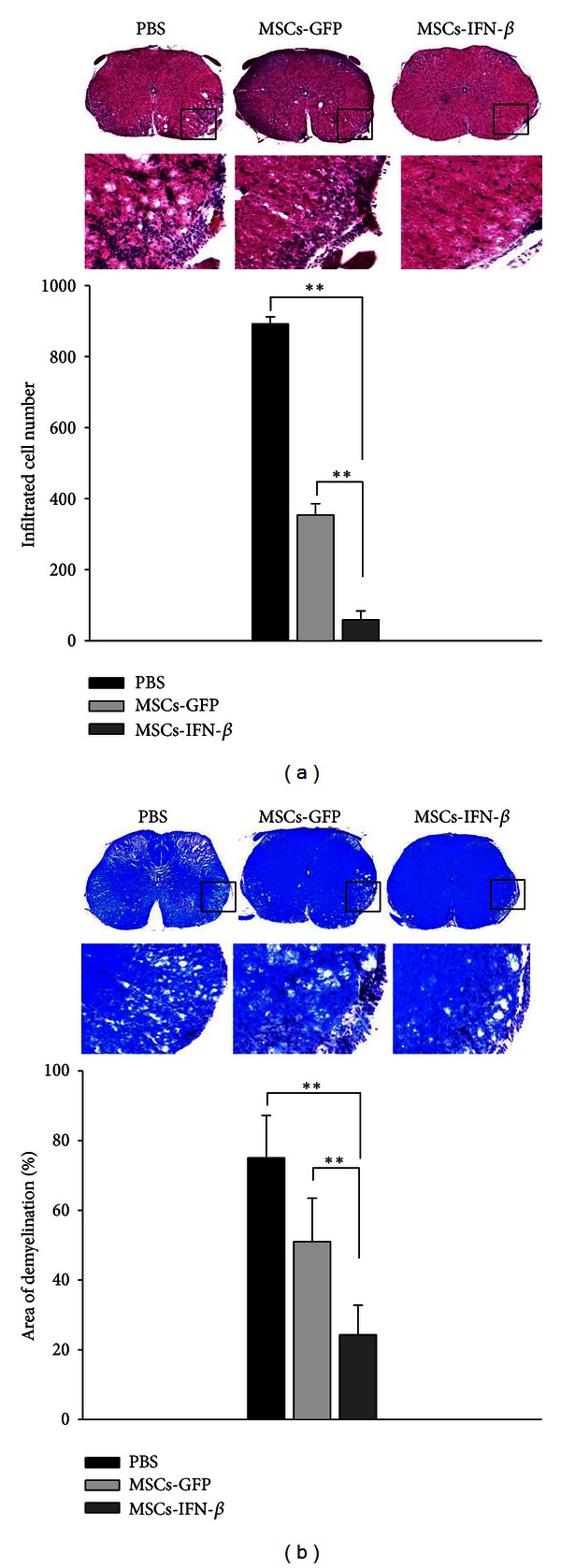
MSCs-IFN*β* reduced inflammatory infiltrates and demyelination in the spinal cord of EAE mice. (a) The representative sections depicting the inflammatory infiltrates in the lumbar spinal cord of EAE mice by each treatment (*n* = 4/each group). Statistical results showed that MSCs-IFN*β* treatment significantly reduced the number of infiltrating cells, compared to PBS- or MSCs-GFP. (b) The representative sections depicting the demyelination in the lumbar spinal cord of EAE mice by each treatment (*n* = 4/each group). Statistical results showed that MSCs-IFN*β* treatment significantly reduced the area of demyelination, compared to PBS or MSCs-GFP treatment. The box represents areas of demyelination and infiltration. Magnification: ×200. Columns: mean; bars: SE. **P < 0.01 and Student's *t*-test. The results are representative of three independent experiments.

The hallmarks of MS include multifocal perivascular mononuclear inflammatory infiltration in the CNS, oligodendrocyte loss, and demyelination [1]. The most important histopathological changes in animals with EAE were detected in the lumbar spinal cord. H&E staining revealed an apparent infiltration of leukocytes into a white matter of the lumbar spinal cord in the PBS- and MSCs-GFP treated EAE mice, but this inflammatory infiltrates were significantly reduced by MSCs-IFN*β*. The number of infiltrated cells in three sections from each of the three animals in each treatment group was counted. A statistically significant decrease in the infiltrated cells into the spinal cord was observed in the MSCs-IFN*β*-treated group compared to the PBS- and MSCs-GFP treated EAE mice (Figure 3(a)). Next, the presence of demyelination was evaluated by LFB staining. Corresponding with inflammatory infiltrates, MSCs-IFN*β* significantly inhibited demyelination in the white matter of the lumbar spinal cord of EAE mice (*P* < 0.01) (Figure 3(b)). These results suggest that MSCs-IFN*β* could effectively improve histological outcomes via reduced inflammatory infiltrates and demyelination in EAE.

### 3.3. MSCs-IFN*β* Modulated the Levels of Th1 and Th2 Cytokines in the Serum of EAE Mice

**Figure 4:**
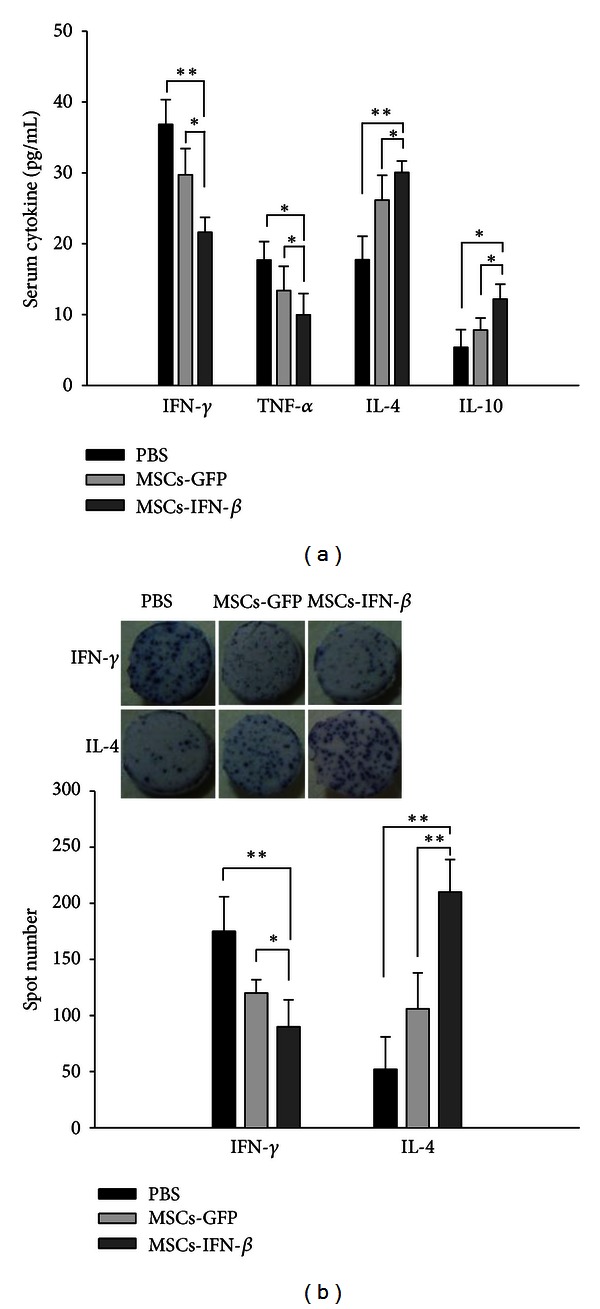
MSCs-IFN*β* modulate immune responses in EAE mice. (a) The serum was isolated from the three groups of EAE mice 30 days after PBS-, MSCs-GFP, or MSCs-IFN*β* treatment (*n* = 3/each group). Cytokine level of Th1 (IFN-*γ* and TNF-*α*) and Th2 (IL-4 and IL-10) in the serum was quantified by ELISA. (b) Splenocytes (5 × 10^5^/well) isolated from EAE mice at day 37 after immunization (*n* = 3/each group), and then the cells were stimulated with MOG35–55. The number of MOG-specific IFN-*γ*/IL-4 producing splenocytes was determined by ELISPOT assay. Representative well show spots for IFN-*γ*/IL-4 production. Magnification: ×1. Columns: mean; bars: SE. *P < 0.05; **P < 0.01; Student's *t*-test. The results are representative of three independent experiments.

To determine whether the MSCs-IFN*β* show immunomodulatory effects on EAE, we evaluated levels of Th1 (IFN-*γ*, TNF-*α*) and Th2 (IL-4, IL-10) cytokines in the serum of EAE mice after MSCs-IFN*β* treatment during the relapsing phase at day 30 after the EAE induction. Cytokine levels of IFN-*γ* and TNF-*α* were significantly reduced in the MSCs-IFN*β* treatment group compared to the PBS- or MSCs-GFP treatment groups (*P* < 0.05). However, levels of IL-4 and IL-10 were significantly increased in the MSCs-IFN*β* treatment group compared to the PBS- or MSCs-GFP treatment groups (*P* < 0.05) (Figure 4(a)). In addition, we confirmed the production of IFN-*γ* and IL-4 in anti-MOG35–55 stimulated splenocytes after MSCs-IFN*β* treatment. The number of MOG-specific IFN-*γ*/IL-4 producing splenocytes was determined by ELISPOT assay. Stereological analysis revealed a significant decrease in IFN-*γ* and a significant increase in IL-4 secretion in the MSCs-IFN*β* treatment group compared to the PBS- or MSCs-GFP treatment groups (*P* < 0.01) (Figure 4(b)). These results indicate that MSCs-IFN*β* systemically affect the effector phase of the disease. Collectively, it appears that there is a significant shift from the Th1 to the Th2 cytokine balance in the MSCs-IFN*β* treatment of EAE mice, compared to PBS- or MSCs-GFP treatment groups.

### 3.4. MSCs-IFN*β* Induced Neurotrophins in the Brain of EAE Mice

**Figure 5:**
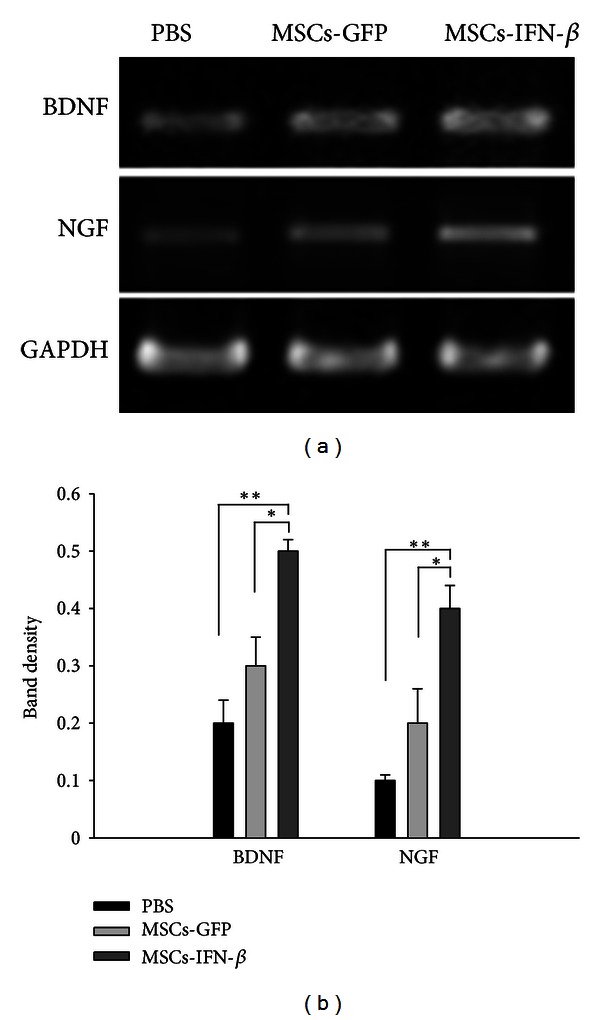
MSCs-IFN*β* induce neurotrophins in the cerebral cortex of EAE mice. (a) RT-PCR analysis for BDNF and NGF in the cerebral cortex of EAE mice after PBS-, MSCs-GFP, or MSCs-IFN*β* treatment (*n* = 3/each group). (b) The BDNF and NGF mRNA expression was significantly induced in the brain of MSCs-IFN*β* treatment group compared to the PBS- or MSCs-GFP treatment groups (*n* = 3/each group). Columns: mean; bars: SE. *P < 0.05; **P < 0.01; Student's *t*-test. The results are representative of three independent experiments.

The beneficial effects produced by MSCs-IFN*β* treatment might be attributed not only to immunomodulatory activity but also to neuroprotective effects. The neuroprotective aspect of inflammation has been documented and is thought to be mediated by neurotrophins (NTs). NTs, such as brain-derived neurotrophic factor (BDNF) and nerve growth factor (NGF), have a dramatic effect on neuron and oligodendrocyte survival, stimulating axonal regeneration and remyelination, and have been reported to be reduced in EAE [18]. Furthermore, some of the approved therapies for MS such as IFN-*β* and glatiramer acetate could exert their beneficial effects by affecting these two NTs in EAE mice. To address this possibility in our experiment, we investigated the mRNA expression of BDNF and NGF in the brain of EAE mice after MSCs-IFN*β* treatment. The transcription level of BDNF and NGF was dramatically higher in MSCs-IFN*β*-treated mice than that in PBS- or MSCs-GFP-treated mice (*P* < 0.01) (Figure 5). It has been reported that IFN-*β* was a potent promoter of NGF production by astrocytes and could induce BDNF production of peripheral blood mononuclear cells in MS patients [19]. Furthermore, NGF has immunomodulatory action on the balance between Th1 and Th2 cytokines, while Th1/Th2 immune deviation plays a pivotal role in chronic progressive and relapsing-remitting EAE [20]. Thus, IFN-*β* secreted from MSCs-IFN*β* could effectively modulate the balance of proinflammatory (Th1) and anti-inflammatory (Th2) cytokines by inducing NTs in EAE mice.

### 3.5. MSCs-IFN*β* Migrate Inflamed Brain and Spinal Cord and Regulate BBB Permeability

**Figure 6:**
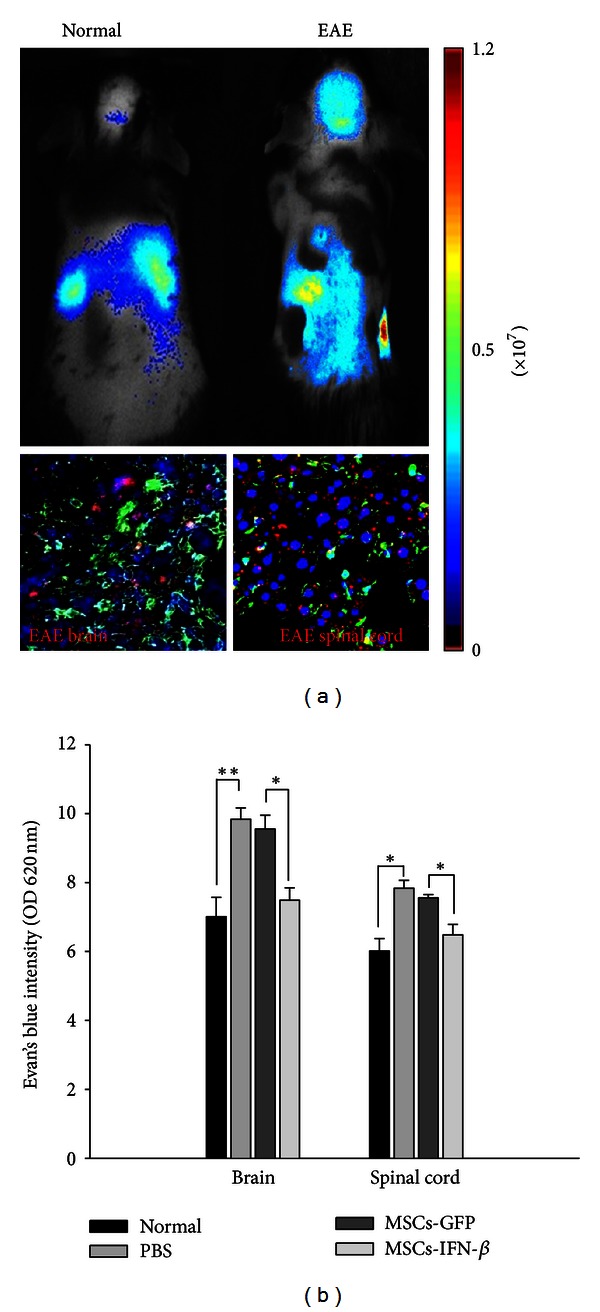
The effect of MSCs-IFN*β* on migration and BBB permeability in EAE mice. (a) NIR-labeled MSCs-IFN*β* detected in the brain and spinal cord of EAE or normal mice at day 7 after treatment (*n* = 3/each group). Mouse images show the biofluorescence signal from the brain and spinal cord detected in one representative animal. The biofluorescence signal is given as photons per second. For the detection of the NIR-positive cells (red) in the brain (bottom-left panel) and spinal cord (bottom-right panel) tissues, cryosections from EAE mouse were stained by human nuclear antigen (hNA). hNA-positive nuclei (green) were stained, and counterstaining was conducted with DAPI (blue). Magnification: ×200. (b) The integrity of BBB of different treated animals was detected by quantitative measurement for EB content at day 7 after treatment (*n* = 3/each group). The intensity of EB in the brain and spinal cord tissue was measured at 620 nm using a spectrofluorometer. Columns: mean; bars: SE. *P < 0.05; **P < 0.01; Student's *t*-test. The results are representative of three independent experiments.

In previous studies, we demonstrated that MSCs have a strong migratory capacity toward injury site and that genetically modified MSCs also have tropism for injury area and improve neurological functional recovery of neurodegenerative diseases [7, 14]. To test this in MSCs-IFN*β*, *in vivo* migration assays using *in vivo* image analysis system. In normal animals, IV injection of MSCs-IFN*β* results in their accumulation in the lung and liver, whereas in EAE mice, the biofluorescence signal is prevalent in areas corresponding to the brain and lumbar spinal cord. Tissue sections from EAE mice also were confirmed by immunohistochemistry at 7 days after MSCs-IFN*β* treatment. Photon emission detected by the Maestro *in vivo* imaging system arose from the presence of NIR labeled MSCs inside the tissues as demonstrated by the detection of hNA-positive elements (green) inside the brain and spinal cord (Figure 6(a)). This result demonstrates that MSCs-IFN*β* also have migratory properties toward the inflamed CNS and could cross the BBB. Inflammation, demyelination, and BBB breakdown are the most common descriptive events in MS. In our experiment, the permeability of BBB of different treated animals was detected by quantitative measurement for EB content at day 7 after treatment. PBS-treated EAE mice showed a significant increase in the content of EB in the brain and spinal cord when compared with the normal control mice (*P* < 0.01). This result is consistent with the previous reports [21, 22]. Enhanced BBB permeability accompanying with large numbers of lymphocytes infiltrating into CNS plays an important role in the development and progression of MS and EAE. However, MSCs-IFN*β*-treated EAE mice showed a significant decrease in the content of EB in the spinal cord when compared with the MSCs-GFP-treated EAE mice (*P* < 0.05). The results showed that MSCs-IFN*β* treatment could prevent further injury of BBB permeability (Figure 6(b)). Reduced BBB permeability may suggest decreased lymphocytes infiltration into CNS then consequently reduced CNS inflammation and demyelination, which was in coincidence with the results of our histological evaluation in MSCs-IFN*β*-treated EAE mice.

## 4. Conclusions

Many studies have been conducted in an attempt to verify the safety and efficiency of various stem cell therapies. hBM-MSCs autografts were one of the first successes in stem cell therapies as there is minimal chance of immune rejection due to their immunomodulatory properties [23]. In addition, these MSC transplantations do not typically result in teratoma formation when tested in clinical trials and are relatively safe compared to ESCs (embryonic stem cells) and iPSCs (induced pluripotent stem cells) which readily form teratomas [24]. Recently, MSC-based therapies have also been evaluated for their safety and varying levels of effectiveness for treating various neurodegenerative disorders, including MS [25, 26]. However, many questions remain regarding the true efficacy and precise mechanism of action of stem cell-based therapeutic approaches in MS. Further clinical trials will need to be carried out to verify the therapeutic efficiency and safety of MSC-based therapies in humans. In conclusion, this study demonstrated that hBM-MSCs can be used as a new delivery vehicle for IFN-*β* therapy against EAE, and MSCs-IFN*β* exhibit strong therapeutic effects by decreasing inflammatory cell influx, suppressing demyelination and immunomodulatory effects by promoting a shift from the Th1 to the Th2 cytokine balance, stabilizing the BBB and preventing the progression of disease in EAE mice. Further studies are needed to identify the detailed mechanisms of therapeutic effect by MSCs-IFN*βin vivo*.
